# Verbal Auditory Cueing of Improvisational Dance: A Proposed Method for Training Agency in Parkinson’s Disease

**DOI:** 10.3389/fneur.2016.00015

**Published:** 2016-02-17

**Authors:** Glenna Batson, Christina E. Hugenschmidt, Christina T. Soriano

**Affiliations:** ^1^Department of Physical Therapy, Winston-Salem State University, Winston-Salem, NC, USA; ^2^Section on Gerontology and Geriatric Medicine, Department of Internal Medicine, Wake Forest School of Medicine, Winston-Salem, NC, USA; ^3^Department of Theatre and Dance, Wake Forest University, Winston-Salem, NC, USA

**Keywords:** Parkinson’s, dance, improvisation, cognition, spontaneity, balance, function, agency

## Abstract

Dance is a non-pharmacological intervention that helps maintain functional independence and quality of life in people with Parkinson’s disease (PPD). Results from controlled studies on group-delivered dance for people with mild-to-moderate stage Parkinson’s have shown statistically and clinically significant improvements in gait, balance, and psychosocial factors. Tested interventions include non-partnered dance forms (ballet and modern dance) and partnered (tango). In all of these dance forms, specific movement patterns initially are learned through repetition and performed in time-to-music. Once the basic steps are mastered, students may be encouraged to improvise on the learned steps as they perform them in rhythm with the music. Here, we summarize a method of teaching improvisational dance that advances previous reported benefits of dance for people with Parkinson’s disease (PD). The method relies primarily on improvisational verbal auditory cueing with less emphasis on directed movement instruction. This method builds on the idea that daily living requires flexible, adaptive responses to real-life challenges. In PD, movement disorders not only limit mobility but also impair spontaneity of thought and action. Dance improvisation demands open and immediate interpretation of verbally delivered movement cues, potentially fostering the formation of spontaneous movement strategies. Here, we present an introduction to a proposed method, detailing its methodological specifics, and pointing to future directions. The viewpoint advances an embodied cognitive approach that has eco-validity in helping PPD meet the changing demands of daily living.

*When we begin to appreciate that movement is what matters … an idea emerges with the potential to reinterpret existing theories of communal dancing in such a way that we can appreciate the ongoing value of dance as vital art here and now. If we privilege bodily movement rather than matter as the currency of evolution, if we insist upon seeing any and all bodily forms as potentials for movement making, then we can begin to perceive dancing, then and now, as a practice in which humans exercise a distinctive human potential to participate in the ongoing evolution of the universe in human-enabling ways* (1).

## Introduction

A hallmark of human movement is volitional control – the freedom to move easily, automatically, and safely within the changing demands of daily living (2). Clinical signs characteristic of Parkinson’s disease (PD) include rigidity and tremor, hypokinesia, stooped posture, vocal alterations, facial masking, and progressive loss of peri- and extrapersonal use of space (3). These movement aberrations are one of many factors that increase the risk of falling and point to the need for improving fall prevention strategies (4). These disease-related limitations experienced by people with Parkinson’s disease (PPD) increasingly rob them of movement freedom. Quality of life declines as conscious attention and effort in everyday living replace spontaneity of communication and safety in navigation (5).

### The Benefits of Dance for PPD

Among the non-pharmacological treatments available to PPD, researchers have shown group-delivered dance classes to be effective in improving functional gains and quality of life measures (6–10). The number of randomized, controlled trials is limited at present due to the newness of the field of the study. Controlled studies in ballet, modern dance, Argentinian tango, other forms of social and ballroom dance, however, have demonstrated meaningful physical and psychosocial benefits (6, 11). A recent review of controlled trials by Sharp and Hewitt (10) found that compared to no intervention, dance results in statistically significant improvements in Unified Parkinson’s Disease Rating Scale (UPDRS) motor scores, balance, and velocity. While exercise, in general, is beneficial for PPD (12), dance may provide benefit beyond those substantiated in common forms of aerobic exercise (e.g., walking, cycling). Relative to aerobic exercise, for example, dance improves balance and quality of life, as measured with the Parkinson’s Disease Questionnaire 39 (PDQ-39) (10).

Exercise can be defined as “physical activity that is planned, structured, and repetitive for the purpose of conditioning any part of the body” (13). Dance can be defined as a “choreographed routine of movements usually performed to music” [Ref. (8) as cited in Ref. (10)]. Using these definitions, dance is a form of exercise where a series of planned movements are choreographed and practiced to achieve mastery. The addition of music may have specific benefits of its own, including being an auditory cue that helps maintain rhythm and unity among the dancers.

The exact neurobiological mechanism through which exercise benefits the brain is not known, but current evidence suggests that exercise-induced increases in blood flow, trophic factors, and anti-inflammatory cytokines may help protect dopaminergic neurons and synapses, or other neural circuits that interact with them (2, 13). In fact, physical exercise may be important for brain health by creating an environment that facilitates plasticity (14). Thus, pairing physical activity with a cognitively demanding task may facilitate learning or performance of that task (14). Increasing numbers of scientific studies indicate the importance of multiple system interaction and its consequent impact on motor and cognitive impairments in PD (15). Researchers who are investigating processes underlying dance making (choreography) have generated the term “creative cognition” (16). These investigations increasingly reveal the importance of mind–body integration and an understanding of cognitive processes through creative movement training. In this way, the cognitive challenges that dance poses may enhance the benefits of physical activity alone.

Dance is viewed as a unique form of movement training because of its potential to create and express new movement patterns. This helps build a psychophysical sense of “embodied and extended agency” (17). Agency is the capacity of individuals to act in the world autonomously and independently (18). From a phenomenological viewpoint, moving freely and easily relies in part on having a sense of agency (19). Agency implies more than merely experiencing one’s body; rather, it suggests a more “embodied” sense of “I can,” that is, that persons own a body and can act on their own behalf and meet their own needs (16, 17). A sense of agency is basic to quality of life, allowing individuals to synchronize their actions with their intentions (19, 20). In the case of learning dance, movement cues draw the mover’s attention to explore agency through expressive whole body gesture. Meaning arises out of participants’ discovery of new physical expressiveness that impacts positively on their ability to act spontaneously and immediately in relationship to change contexts (21, 22).

### Improvisation Defined

To date, protocols for group-delivered dance classes for PPD largely have employed choreographed dance with rhythmic, musical accompaniment (e.g., modern dance/ballet), or prescribed partnering moves within a codified and widely used social dance system (e.g., tango and other familiar social dance forms, such as ballroom or folk dance). These approaches have shown good success in promoting functional gains in balance, gait, and UPDRS score.

Improvisation is the ability to create new gestures and movements on the spur of the moment (23). Improvisation is not limited to dance but is also part of other performance arts, such as music or drama. Within the performing arts, the definition of improvisation varies (24–28). However, a common conception is that improvisation evokes acting on the unexpected and unknown. The objective is that preplanned or prescriptive movement, copying or mimicking are replaced by the possibility for novel physical responses. Improvisation does not imply that the event lacks structure or that people are free to do whatever they want. While material may be unplanned or unexpected, it is not random (26). Instead, improvisation allows movers to make empowering choices within a structured environment of select constraints (28, 29). In this way, improvisation is directly linked with the idea of agency, of a person’s ability to act autonomously.

### Improvisation as a Form of Dance

Although many dance forms may appear stylized or systematized, they can include elements of improvisation. For example, Argentinian tango is a form of social (predominantly partnered) “street” dance that allows for improvisation within its codified form. However, improvisational movement alone can be taught as dance, one that offers a distinct form of movement variability, in and of itself. This form of improvisation may have unique benefits for the PD community, benefits that await substantiation through comparative research.

Two key differences distinguish teaching improvisational dance as the primary dance form from encouraging improvisation around another form of dance. These are (1) the use of auditory cueing and music and (2) repetition of choreographed or codified dance movements. In improvisational dance, verbal auditory cueing (VAC) is used to convey improvisational ideas that elicit novel movement from students in the class, even at the earliest stages of learning. Because the VAC does not instruct explicit movements, participants self-select motor strategies in response to the prompt that vary in terms of shape of movement, spatial usage, and timing. Unlike many other forms of dance or exercise, improvisation is not learned by repetition and reinforcement of specific steps.

In addition, in improvisational dance, movement may or may not be rhythmically synchronized with the music. This is an important point because auditory cueing in the form of rhythmic tones has been shown effective in normalizing gait impairment in PPD (30). The presentation of tones can be as simple as rhythmic beeps, and benefit is often limited to when the tones are played. Regardless of delivery (teacher, caregiver, or affected person), cues help initiate, sustain, and terminate movement as tasks demand. In choreographed solo or partnered dance, the strong rhythmic musical beat arguably is essential in cueing timing of the basic steps needed for accuracy and safety in the initial stages of learning. Rhythmic music has been shown to be an effective form of auditory cueing in dance instruction in partnered Argentine tango (7). It is clear that the benefits of choreographed and partnered dance are not limited to moments when music is playing; yet, there may be a specific benefit to cue movement initiation without a rhythmic cue.

### Previous Studies of Improvisational Dance in PPD

To the best of our knowledge, two small clinical studies have been conducted specifically evaluating improvisational dance as an intervention for PPD and both have shown select improvements in balance over relatively short time spans (31, 32). Pilot work by Marchant and colleagues (32) showed statistically significant improvements in the UPDRS motor score, Berg balance scale, and features of gait (increased swing/decreased stance) in a 2-week intensive workshop in a specific form of improvisation known as “contact improvisation.” In 2014, our group added to this nascent field with a pilot study of a 7-week improvisational dance class (31). Statistically significant improvements were observed in balance on the Fullerton Advanced Balance Scale, and improvements of a clinically significant magnitude were obtained on the Timed Up and Go. In addition, in a subsequent case study (31), resting-state functional magnetic resonance imaging of one participant was performed and a graph–theory analysis of community structure was performed. Briefly, community structure analysis shows what brain regions are more functionally connected with each other than with the rest of the brain, revealing brain “neighborhoods.” As an example, the occipital lobe is often identified as a community or neighborhood in this analysis, as the visual cortex is more connected with itself than with the brain as a whole. For more detail on this method, please see Ref. (33). Before the intervention, it was observed that the basal ganglia were in a community that only consisted of the basal ganglia. That is, the basal ganglia were more connected with themselves than the brain as a whole. After the intervention, the community structure of the basal ganglia had changed, and now included premotor cortex. Now, the basal ganglia and premotor cortex were more functionally connected to each other than shown at baseline. While these studies are small, with neuroimaging data only available for one person, these results suggest that improvisational dance can result in meaningful changes in movement and perhaps also brain networks.

### The Potential Relevance of Improvisation for Automaticity in PPD

Daily life demands a high degree of automatic functioning. This means employing flexible, adaptive strategies to alter conditions, or switch tasks between automatic (unconscious, habitual) and goal-directed (conscious, volitional) movement (13). Effective switching between these modes of movement allows for flexibility and automatically to another task, but rapidly to recruit conscious control if the environmental demands suddenly change. Unanticipated events call upon the person’s ability to create motor strategies in the moment in response to balance perturbations and challenges to planning, problem solving, and memory.

Automaticity of gait and balance are both affected in PPD (30). The hallmark of PD is the deterioration of dopaminergic neurons in the basal ganglia, a group of subcortical brain structures that are central to movement, learning, and motivation. Recent research suggests that the basal ganglia are topologically organized into functional divisions (34). The sensorimotor portion of the basal ganglia, the region first affected by PD, is characterized by high connectivity with sensory and motor areas of cortex and is thought to be key in maintaining automaticity of movement (13, 34, 35). The enhanced connectivity between the basal ganglia and premotor cortex seen in response to improvisational movement supports the idea that improvisational dance may be possibly be changing brain networks involved in automaticity in meaningful ways.

Because the loss of automaticity requires movement to be guided by conscious control, cognition is also taxed when automaticity deteriorates. PD has been shown to negatively alter automaticity, altering speed variability and quality of gait (locomotion), particularly in dual- and multi-tasking conditions (36). The ability to restore automaticity could therefore benefit both movement and cognition in PPD. Preliminary evidence is emerging that the use of contemporary dance improvisation may improve cognitive flexibility in normal aging populations (37). Here, measurable improvements have been shown in executive functions such as attentional control and goal directedness in the face of distracting activities and ability to switch tasks on cue (38). Improvisational dance builds on perceptual awareness of the moving body in a moving context (that is, a context in which other people also simultaneously are moving in the same space). This constantly changing context challenges balance, agility, attention, choice and decision-making, and other physical and cognitive skills.

Following this reasoning, one might expect that for someone with good automaticity (automatic movement responsiveness), the prefrontal cortex, which is highly linked with conscious cognitive control, would be in a different network neighborhood than the motor cortex. That is, not to say that those brain networks would not interact, but rather that they would not be preferentially connected. In someone with poor automaticity, who often relied on prefrontal cortex to compensate for losses in automatic movement control, prefrontal cortical activity might be expected to positively correlate with motor cortical activity, at least during certain tasks. Interestingly, neuroimaging literature on improvisation suggests that frontal cortex may be deactivated during improvisation, while motor and premotor regions are more active (39–41), suggesting that improvisation might train the motor and premotor regions to operate independently of prefrontal regions. In other words, improvisation may help to train brain networks involved in automaticity.

## Methods

Reported here is one dance teacher’s (Ms. Soriano) method in teaching dance to mild-to-moderate stage PPD over a 3-year period (2013–2015). Ms. Soriano is a tenured university dance professor also trained in the Mark Morris Dance for PD^®^ program. Ms. Soriano developed her method by engaging in several pilot studies at her home university and teaching a community class series over this time frame. She has taught the method to medical professionals and artists and will continue to do so. She is trademarking the name IMPROVment for this method. In addition to the information provided here, introductory details regarding the method are posted at www.improvment.us. Detailing the method will serve as a model class structure that other dance and movement instructors can consider in designing movement classes for communities of PPD. As well, the model can be used by medical professionals, such as physical and occupational therapists, to evaluate the feasibility of IMPROVment exercises for specific groups of patients or types of care.

The primary aim of the improvisational movement approach described here is to propose a method of physical problem solving that enables students to develop more positive responses to psychophysical challenges faced in everyday living. The improvisational class structure explained here aims to promote the sense of agency needed to navigate independently, efficiently, and safely in daily living by promoting physical responsiveness. The method does not seek primarily to improve select functional outcomes (e.g., balance) or to employ compensatory strategies as a means of coping with dysfunction. Rather, the focus is on movement potential for PPD, the ability to generate new movement strategies. This affords a new vision of physical independence, one who assumes greater relevance as aging accompanies neurodegenerative illness.

### Description of Students/Participants

Participants in the original pilot study (31) were assessed as having mild-to-moderate PD with Hoehn and Yahr scores between 1.5 and 3 and their carepartners. For the ongoing community classes, participants are referred by their neurologist or through the Winston-Salem community support group and represent a range of both physical and cognitive function. In addition, one class has been taught to non-PD older adults who signed up for the class as a continuing education opportunity. Prospective students are invited to watch the class the first time and are asked to sign a waiver before active participation begins. The class includes both PPD and their carepartners, who may be spouses, family members, or paid caregivers. Some class members use assistive devices to walk.

### Description of Class Setting

Classes take place at a private dance studio in a central location of Winston-Salem, NC, USA. There is ample parking within 25 ft of the studio entrance. The studio affords stair-less access, with handicap bathrooms <20 ft from the center of the studio space. There is plenty of ambient light and all environmental obstacles are removed or otherwise controlled. Chairs are available and are arranged in a circle in the middle of the studio at the beginning of class. Two walls contain a ballet barre and mirrors, and a moveable barre is located along a third wall. The class lasts approximately an hour and is free of charge.

### Theory and Philosophy

From a theoretical perspective, the IMPROVment method finds its complement in embodied cognitive science. Embodied cognition states that bodily movement plays a constitutive role in agency and thought (42–44). The ability to act on one’s own behalf efficiently and effectively does not come from mental computations alone but from direct experience of body movement within varied exposure to environments and tasks.

IMPROVment classes are grounded in a series of four core principles that shape the overall tone of the class and result in a sense of community and social belonging: non-judgment, non-competitiveness, curiosity and playfulness, and risk taking. Importantly, for implementing this method, the teaching vocabulary (the selection of verbal auditory cues) issues from these core beliefs, many of which are central to the spirit of dance improvisation. These are presented in Table 1 and described in detail below.

**Table 1:** **Principles of improvisational dance and methods for their inclusion in class**.

Principle	Methods of instantiation
Non-judgment	Class advertised as movement class
	Greeting at entrance by teacher
	Class offered free of charge
	Inclusion of carepartners as students
	VAC that there are no mistakes, only new movement options
Non-competitiveness	All movement is seen as an honest effort
	VAC focused on action, not quality of movement
	VAC “Yes, and …” replaces “Not that” or “Rather try this”
Curiosity and playfulness	Awareness of movement possibilities
	VAC “Stay curious in what you are doing” or “Keep going”
	VAC “Nothing is precious”
	Pacing of VAC does not allow self-editing
Risk taking	Selection of class environment
	Student self-selection of participation level
	VAC to validate self-selected level of participation
	Adaptation of exercises to sitting or at the barre as needed
	Structured, directive (non-improvised) activity transitions
	VAC to attend to constraints of an activity rather than invoke fear

#### Non-Judgment

The ability to move without judgment of self or others may challenge group members. Many perceived fears (real and imagined) present barriers to social participation for PPD (45). A space is created where PPD enter into significant camaraderie, look after one another, and form deep friendships. Class participants might regularly see one another in various community support group settings. However, dance class goes further in encouraging a sense of inclusion as participants are brought into physical play without judgment.

Generating an atmosphere of non-judgment is an active process. Concern for both individual and the group is consciously established from the first introduction to a participant and carried through the class. The class is advertised as a movement class instead of a “dance” class to encourage participation of those who think they cannot dance. Classes are free of charge to encourage participation across economic sectors. Care partners or spouses are strongly encouraged to join. Students are explicitly instructed that there are no mistakes or “better” ways to move – only new movement options.

Peer witnessing plays a part in generating group enthusiasm, inclusion, and acceptance. The class enables participants to see each other “perform” – that is, letting go of self-consciousness and becoming spontaneously expressive through their whole bodies. Some of the exercises are done with eyes closed to help participants “convene with their own bodies.” This cue helps support trust in the rightness of each person’s own movement choices. Participants appear to overcome their anxiety to perform well and simply become themselves, each of who is perceived as a unique character in the colorful mix of group expression. Thus, participants are seen and received “differently,” as “expressive” or “creative.” Dance improvisation engenders a different layer of sociability as persons are witnessed as being “funny,” “endearing,” or “dramatic.”

#### Non-Competitiveness

A related core value in IMPROVment is that everyone can dance in some way regardless of age, stage, and condition. The overriding tone of the class is that all movement is an honest effort. The degree to which one moves and the type of movements expressed are of equal value. Movement cues focus on action, on generating movement itself, without value placed on quality of movement. Rather than attempting to move like someone else or move according to a preconceived ideal of rightness or normalcy, the focus is on learning to simply move for oneself, whatever the outcome.

The phenomenological experience of agency, the possibility and potential of “can do,” underscores confidence, self-efficacy, self-acceptance, and empowerment. Improvisation is introduced as a palette of options or choices that are not final solutions, but possibilities for more choices. An auditory prompt can result in an infinite number of movement ideas and variations. The pre-reflective, non-planned awareness of the body moving (improvising) in space is each participant’s unique and empowering form of agency. As participants come up with one movement choice, they are encouraged to choose another and another. “Yes, and …” is the cue, as opposed to saying “No, not that …” or “rather … try this.”

#### Curiosity and Playfulness

Exerting agency requires “active” curiosity. This means that participants become more aware of the various shapes and movements the body makes in space and in relation to others. Curiosity is emphasized over competitiveness. Participants simultaneously invest in what they themselves are doing while staying curious about what might happen next. Attention is drawn to keep pace with VAC within the changing movement conditions. Prompts enable self-generated movement that continues in real time without extra time to reflect, self-edit or otherwise change the original choice made. These movement cues themselves induce curiosity. The prompt to “keep going” (with one’s movement choice) is synonymous with the prompt to “stay curious and interested in what you are doing.” Playing the game often supersedes self-consciousness. Participants may not be able to keep up with each and every cue. They can, however, stay intensely focused on and motivated to the present task, rather than becoming inhibited. Every person is invited to share (try on) gestures that created by others in the class with a playful spirit, as opposed to feeling pressed to perform. “Nothing is precious” is a repeated VAC, reminding participants that each spontaneous movement made is part of the ongoing group field of play.

#### Risk Taking

Physical risk taking is encouraged within reasonable margins of safety. Increasing one’s ability to take risks when moving is integral to the class structure in order to maximize the effects of improvisation. Feeling safe helps participants challenge themselves and promotes autonomy and a sense of personal agency. As participants become more comfortable and confident in responding on cue, they usually react to new instructions more quickly, moving with increased speed and demonstrating greater diversity and flexibility of physical responses.

As described in Section “Description of Class Setting,” the class location and environment are designed to maximize safety. In addition, strategies are used in class to help maintain safety while facing various balance challenges. First, the IMPROVment method encourages each member of the class to self-select his or her participation level. As an example of self-selection, persons may not be able to carry out complex locomotor movements and choose to adapt these sitting or at the ballet barre. Participants receive frequent VAC reinforcing their self-selected choices. This allows for support of all class members, regardless of level of ability, physical limitation, or apprehension. Self-selection helps reinforce not only autonomy and personal agency but also safety.

Second, transitions from one phase of the class (e.g., seated) to another (e.g., barre) are monitored carefully for safety. Transitions are guided, as opposed to fully improvised. Care partners or registered health care assistants who take the class with their spouse or family member living with Parkinson’s, assist at these times as needed. Ms. Soriano and a trained undergraduate assistant may “shadow” class members to and from their chairs or the barre. Velocity changes are common in these transitions. The ability to control velocity changes can depend on the ease of recruiting smaller joint sub-movements to execute task changes. This has been demonstrated in reaching tasks for this population (46). It is important to watch a participant’s ease of movement from one movement from sitting to standing, or from one phase of the class to the next (from chairs to the stationary ballet barre, or from the barre into the center of the room).

Third, VACs train group attention to the constraints of the task and the environment, rather than evoking fear reactions. Rather than warning participants to “be careful” so they would not fall, the statement “Be aware of your surroundings and others moving simultaneously in the room” helps keeps everyone consciously aware of the task context.

Fourth, increasing challenge is introduced by gradually increasing the variety and complexity of movement tasks. This is important in learning to be safe while increasing the embodied sense of confidence.

### Training Strategies

Every moment presents multiple challenges in terms of balance, awareness of space and environmental constraints, awareness of self and others within the space, requiring a response that integrates the brain and body, and automatic and intentional movement. Ms. Soriano uses the following main training strategies to maintain a level of challenge for cognition and physical activity: active imagination, variability, and pacing.

#### Active Imagination

Working with imagery is crucial in an improvisatory practice. VACs are used to create movement scenarios that cue or activate the motor imagination. VAC takes primacy over rhythmic entrainment to music, although the music itself may be used as an improvisatory cue. The teacher calls the cue, demonstrating an optional response, and asks participants join in immediately with their own gestural inventions. As an example, students might be prompted during the seated warm-up to recreate a beach scene. VACs direct motor imagination by using rich language to act out a beach scenario – laying down their beach blankets, putting on sunscreen, opening their picnic baskets and setting out lunch on a blanket, running into the ocean, avoiding the shark swimming toward them, and so forth. Often, participants will do this seated, with eyes closed. This activates the imagination more strongly and adds a balance challenge.

#### Variability

The IMPROVment method does not aim to learn a specific movement pattern and habituate to it. Rather, the aim is to intend to stimulate new pathways for motor learning by meeting unexpected environmental conditions arising in the moment and devising new physical solutions as a result. Daily life is fraught with the unexpected, with variable environmental encounters that call for ongoing problem solving. The variability inherent in improvisational movement training helps minimize the tendency for people to default to their habitual (familiar) ways of moving and consider new movement strategies. Preliminary research suggests that that variability acts as a novel stimulus to the motor cortex, facilitating new motor pathways (38).

Cues often are delivered quickly, one after another, to increase excitability of the motor cortical as well as enable participants to go beyond their habitual (“self-perceived”) capability (13). Within an average of 2 min, tasks requiring quicker decision-making are implemented. Physical challenges are advanced by dual- and multi-tasking, such as being asked to direct traffic with the right side of the body while picking apples with the left.

Maintaining variability throughout the class can be a challenge. Variability is accomplished by adding complexity, presenting new prompts, or assigning a qualitative change to a specific exercise. For example, students might be given a simple prompt to make any shape with their upper body. Variability can be added by increasing complexity, e.g., a VAC to create a series of new shapes, each one different from the previous, and then to remember and reproduce the first shape. Variability can be added by presenting a new VAC, e.g., “Now make a second shape with your lower body.” Variability can also be added by assigning a qualitative change, e.g., a VAC to swim with the upper body and lunge with the lower body. Variable movement themes (e.g., changes in body shape and movement direction) are intended to stimulate movement inventiveness and to avoid defaulting to habitual responses.

Improvisational prompts challenge the scope and speed of movement by introducing multiple body parts engaging in multiple tasks. For PPD, maintaining variability of movement becomes a particular challenge when cueing dual- and multi-tasking activities (36).

#### Pacing

Pacing refers to the rate at which new movement prompts are presented. As with variability, quick changes in pace also avoid defaulting to habitual responses, thereby facilitating new movement options. Participants cannot rely on copying another, or necessarily their own memory or anticipation for the answer to the motor problem, Verbal cues might be delivered in a rapid-fire manner, for example, in order to keep participants from having the time to think or reflect on movement choices. There is little time to change one’s mind, become embarrassed, or be dissatisfied with the choice made. Participants simply need to move through the chosen sequence, even if it might not seem logical or conform to their ideal conception of the movement. Even if the outcome does not match their intention, participants often surprise themselves in seeing that they are capable of new movement choices.

### Music Selection

Participants are invited to bring music in for class. Music is used in different ways throughout the class. The playlist is random and variable, not the source of rhythmic entrainment. Sometimes, it is unrelated to movement instruction and is relegated to being ambient, such as something that might be heard at a cocktail party. Other times the music may serve as an improvisational cue. As examples, students may be asked to dance what comes to their minds listening to Otis Redding’s “(Sittin’ on) The Dock of the Bay” and then variations are cued from that initial movement, or “play” an instrument they can hear in a complex piece of classical or jazz music. Sometimes, participants themselves create movement through vocalizing or body-based percussive actions. Portions of the class also happen in silence. Video analysis of previous studies shows that participants are more likely to improvise rather than follow (entrain to) the beat of any music played.

### Class Structure

The general class structure supports the core philosophies of IMPROVment and has four phases. These include (1) group warm-up in chairs positioned in a circle, (2) standing barre with solo- and partnering exercises, (3) moving as a group through free space (with and without a partner), and (4) recuperation and rest.

The exercises in the IMPROVment method are designed to help PPD engage with the challenges of motor control and coordination. The exercises present a movement theme for each class and increase in complexity as the class progresses. For example, an exercise practiced in chairs might also reappear at the barre, challenging participants to interpret in standing a prompt previously given in sitting. An example of an exercise that might thread throughout class is what Ms. Soriano refers to as “Out Out, In In” (OOII). OOII asks for inventive and varied examples of distal extension and contraction of limbs (including the head) either toward the center of the body (proximally) or away from the body center (into space). This general objective invites a diversity of expression, speed, and options and is incrementally more challenging from seated to ambulating. Examples of OOII in each of these setting are detailed within the four phases in Table 2. Regardless of physical ability, participants are able to participate in OOII in multiple expressive and meaningful ways, even if never moving from the chair.

**Table 2:** **Demonstrating the tier system through one exercise: Out Out In In (OOII)**.

Position/phase	Purpose	Challenge	Motor imagery cues	Pacing
Seated warm-up (10 min)	Testing seated balance by proximal-to-distal gesturing and weight shift	Reaching and shifting weight beyond base of support	Imagine a beach: “Ouch! The sand is hot! Rush to the ocean with the biggest steps and reaching movements you can make … Now, you’re in a boat, fiercely rocking in a storm … Don’t fall out!!”	Slow, deliberate-to-fast traveling movement
Stationary partnering at ballet barre (5–10 min)	Testing standing balance in a stable environment	Creating near-fall conditions within margins of safety (barre always within reach)	“The barre is your partner – trust you can support with one hand as you reach for the apple from the highest tree branch. Now, slow dance with your ‘partner’ … cheek-to-cheek’ … now switch to the Lindy” (jitterbug)	Languid stretching of the center (spine) evolves to large, dynamic reaches in space
Center floor walking	Testing balance in a mobile environment with and without an obstacle course (chairs)	Quick decision-making. Stopping and starting at will	“Walk in the space … now pause. Now, every time I clap, change direction. Now when I clap twice, make a shape close to your body; Clap-clap – now, the largest shape you can imagine!”	Slow-to-fast gesturing as verbal commands challenge unanticipated changes of direction and body movement
Center floor partnering	Mirroring exercises	Leader-follower responsibility with the challenge to listen, hear, and replicate another person’s actions	“Mirror your partner’s movements as though you are moving underwater.” “Mirror ‘angular’ movements … now ‘flowing’ movements. Now combine both qualities”	Pacing again variable and unanticipated as the play between two partners evolves

Similar exercises may appear within each phase creating a ­progression of motor skills, but the class series does not build incrementally. Throughout the class, there are recuperative phases that are essential not only to rest and recover metabolically but also cognitively. These movements are slower, simpler, and often more familiar. For example, simple familiar exercises are introduced such as a seated hamstring stretch. Here is the opportunity to let go of attending to task and enjoy more automatic movement patterns. Material varies between phases, helping to consolidate memory (40) without using repetition as a means of reinforcement. Concepts and tasks are heterarchical and interdigitated. VACs used in class are open-ended. That is, they do not provide a movement solution, but rather a cue that has multiple movement solutions. Below, each phase is described in more detail and an example exercise is presented.

#### Group Warm-up

The initial phase of the class takes place with participants seated in chairs where their sitting balance is tested by a variety of ways of shifting weight. Prompts demand while moving expansively to the limits of their self-selected range while reinforcing curiosity, imagination, and playfulness. Even while sitting, rich imagery is used, pacing remains quick, and variability is rapid. Seated exercises are still demanding.

A commonly performed seated exercise in this phase is called “Pass the Energy.” “Pass the energy” is designed to invite every participant-to-participate and be empowered by his/her movement choices. Just as it sounds, “Pass the Energy” asks participants to create a gesture with their whole body and “pass” that gesture to the next seated person. Each person’s “energy” has unique qualities and calls for eye contact as the recipient takes the “energy” and transforms it into his or her own version. After participants make their first movement choices, increases in pacing and variability add complexity to the exercise constraints (as described in Sections “Variability” and “Pacing”).

#### Standing Barre

The class usually progresses from seated movement to moving at a ballet barre. The barre is not treated as a “prop” to rely on for securing balance. OOII practiced here, for instance, encourages participants to imagine the ballet barre as a dance partner. With a VAC to reach away from one’s “partner” and then come back to it, participants find novel ways to step out or away from the barre and then come back toward it. Balance is challenged and tested in safe ways while reaching high and low in the space. The VAC “Out Out, In In” encourages the possibility of someone reaching away (or outwardly) from the barre with eventually no support and then returning toward the barre (inwardly) with one or two hands. This not only can be repeated multiple times but eventually, participants also become curious to try turning around themselves as they long for new and inventive ways to practice OOII.

#### Moving through Free Space

Walking variations follow, first as freely moving on one’s own in the space and then interacting with others in the class in partnered and non-partnered interactions.

For example, Ms. Soriano sets up various obstacle courses in the studio by using chairs, requiring gradual and incremental changes in speed and direction at first. The rationale behind this decision is not to create a hazard, but to reflect the improvisational nature of everyday life where obstacles will inevitably happen. Similarly, like the ballet barre, the chair can become another dancing partner. Chairs can become sculpture pieces that participants lift and move around the room with the VAC to consider the design of the chair in the space, with relation to one’s body, other bodies, and other chairs in the room.

Another example of a VAC for moving around chairs in the space is to walk with a certain “effort percentage.” This encourages participants to consider that they are in control of when they start and stop, as well as how fast or slow they move. Whatever the configuration of chairs in the space, participants might be given the VAC, “if the pace with which you are walking around the room now is 40%, transition to what 60% looks like (e.g., 20 or 75%).” Ms. Soriano determines the range of percentage changes in walking speed, based on the average pace the class presents in the moment. Finally, another walking exercise could be the simple VAC to walk and pause in the room over and over again, and each time during a pause period, create a different shape with your upper or lower body. In keeping with the improvisational emphasis on encouraging agency, each participant determines for him- or herself how long one pauses, or how long to walk before pausing.

#### Recuperation

Class closure always involves the ritual of circling up, sharing a common gesture of celebration or applauding each other. Often, this circle opens out to become a “free dance.” Here, participants are encouraged to dance in free form about the room with or without music playing, making eye contact, and gesturing to each person in the class to bring full closure.

## Discussion

This article describes the philosophy, methods, and class structure of one dance teacher’s method in using VAC of improvisational dance movement to enhance agency in PPD. The method is designed first as a tool to expand the range of self-perceived movement potential in persons with early to middle stage PD, and second, to build a caring and supportive environment in which to develop a fuller range of physical options and strategies for movement to meet the challenges of everyday navigation and communication.

Improvisation is not a prescribed set of exercises. Exercises are provided here as examples and starting points with the hope that teachers who wish to use this method will improvise their own exercises and in the future, share them *via* the website (www.improvment.us). One challenge in both teaching and researching improvisation is the fact that exact repetition is explicitly avoided. What is repeated and can be taught, what unifies the method presented here, are the philosophies of non-judgment, non-competitiveness, curiosity, and risk taking; and the teaching strategies of active imagination, rapid pacing, and variability.

### Improvisation and Emotional Well-being

In addition to the physical benefits for PPD documented in the limited data collected thus far, improvisational dance also may benefit emotional well-being. Subjective reporting by participants in IMPROVment classes, indicate improvement in social and emotional well-being. A survey was developed for IMPROVment classes which queried the participants’ ability to act for and by themselves. This survey asked for anonymous feedback at a randomly selected point in the ongoing community class. Questions were rated on a 5-point Likert scale, the scoring of which ranged from 1 “Not at All” to 5 “Very Much.” Previously unpublished responses from the eleven respondents are reported here. The surveys were collected with ethical oversight and approval from the Wake Forest School of Medicine IRB in accordance with the Declaration of Helsinki. Overall responses were positive. Seven of the 11 respondents stated increased life satisfaction “Very Much.” Nine respondents rated the class as 4 or 5 for increased empathy for fellow classmates, creativity and physical benefits, and movement skills. Six respondents noted improvements in balance (4 or 5 rating). Open-ended comments were positive and many focused on social or emotional well-being. Some examples are “increased mobility, more confidence, more energy, helps all the way around”; increased “camaraderie and community” and “social interaction”; “laughing, fun, getting to connect with new friends, body warming up.”

To date, direct comparisons with other dance methods have not been made in this particular sample, however. A 2009 study comparing Argentinian tango with Waltz/Foxtrot and Tai Chi (47) sets a precedent for this, wherein improvements in quality of life (including emotional well-being) allude to the specificity of well-being afforded in dance training. The researchers hypothesized that significant findings were due to addressing balance and gait in the context of closely connected improvisational partnering, characteristic of tango.

### Future Directions

Clinical guidelines of current protocols for teaching any style of dance to PPD are still evolving, and therefore, this article serves to contribute to a growing understanding of safe and effective community-based, group-delivered dance programs for this population.

There is a clear need for additional research using larger sample sizes to examine the potential long-term effects of dance for those with PD (6). Most studies of dance for PD, to date, have used rather small sample sizes and have only examined the short-term effects of dance programs, as have most studies of creativity and improvisation. Future work should include larger samples and assessment of the long-term effectiveness of dance for PD, and optimal dosing of dance interventions with respect to frequency, duration, and intensity. Of particular relevance here would be to assess the effectiveness of VAC both in structured and improvisational contexts, as well as to compare behavioral results with brain mapping data.

While admittedly variable in its breadth and depth, current evidence suggests that aerobic exercise, mind–body exercise such as Tai Chi, choreographed dance, and social dance can all benefit people with PPD. This is good, because the more valid exercise alternatives that exist, the more likely people are to find a movement class that in enjoyable and accessible to them, increasing the likelihood that they will maintain a level of physical activity that benefits their health. One purpose of this article is to advance the idea that improvisational movement should be further investigated as another method of movement instruction that may contribute unique advantages to people with PPD.

## Author Contributions

All authors made equal contributions intellectually and practically to the conception, preparation, and execution of this manuscript with mutual mentoring and support.

## Conflict of Interest Statement

The authors declare that the research was conducted in the absence of any commercial or financial relationships that could be construed as a potential conflict of interest.
